# Dataset on the major and trace elements contents and contamination in the sediments of Saronikos Gulf and Elefsis Bay, Greece

**DOI:** 10.1016/j.dib.2020.105330

**Published:** 2020-02-26

**Authors:** Aristomenis P. Karageorgis, Fotini Botsou, Helen Kaberi, Stylianos Iliakis

**Affiliations:** a Hellenic Centre for Marine Research, Institute of Oceanography, 46.7 km Athens-Sounio Avenue, 19013, Anavyssos, Greece; b Laboratory of Environmental Chemistry, Department of Chemistry, National and Kapodistrian University of Athens, 15784, Athens, Greece

**Keywords:** Metals, Sediments, Saronikos Gulf and Elefsis Bay, Eastern Mediterranean, Factor analysis, Enrichment factors, Temporal trend analysis, Assessment of contamination

## Abstract

Coastal marine sediments receive intensive stress from urbanization and industrialization, which is manifested by increased contents of heavy metals and organic pollutants. Saronikos Gulf and the small embayment of Elefsis, stretch along the coast of the greater Athens and Pireaus port, the most urbanized and industrialized areas in Greece. Here we present the data of a 20-year geochemical record on grain-size, organic carbon, and major and trace elements contents of the Saronikos Gulf sediments. A total of 216 sediment samples were collected within the period of 1999–2018 from the four sub-sectors of the gulf, namely, the Elefsis Bay, the Inner, Outer, and Western (Megara and Epidavros basin) Saronikos Gulf. Additionally, at least one core was obtained from each sub-sector. Sediments deposited at pre-industrial periods were recognized by ^14^C and ^210^Pb dating, and served for establishing regionalized background levels of metals. Factor analysis was conducted to reveal the inter-parametric relationships, thus their common sources, as well as transport and deposition pathways. Then, Enrichment Factors and the multi-elemental Modified Pollution Index (MPI) were calculated to assess the current environmental status of the sediments. Data of sampling sites with at least a five-year record, were assessed for temporal trends, to explore whether sustained, increasing or decreasing trends of the MPI are observed. The dataset and analyses presented here support the research article entitled *Geochemistry of major and trace elements in surface sediments of the Saronikos Gulf (Greece): assessment of contamination between 1999 and 2018* [1].

**Table:** Specifications Table

Subject	Oceanography
Specific subject area	Geochemistry of sedimentary major and trace elements and pollution assessment
Type of data	Table Chart Graph Figure
How data were acquired	Sampling: Box corer, gravity corer; Major and trace elements: X-ray Fluorescence (PANalytical, PW-2400 wavelength dispersive spectrometer); grain-size: Sedigraph (Micromeritics 5100E, Micromeritics III *PLUS)*; OC: CHN analyzer (Fisons type EA-1108);^210^Pb (Ortec EGamp; G));^14^C AMS dating (Beta Analytics Inc.); data statistics (XLSTAT); Trend assessment: MAKESENS software
Data format	Raw
Parameters for data collection	Surface sediment samples (n = 216) were collected over an extended sampling network in order to achieve spatial and temporal coverage of sedimentary geochemical data in the Eastern Mediterranean with respect to major and trace elements, together with grain-size and organic carbon parameters. Sediment cores were obtained from all sub-sectors of the study area to assess the regionalized background levels of metals and facilitate future contamination assessments in the Eastern Mediterranean's regional seas. Statistical analysis was conducted to assess the sources, and transport pathways of the variables studied. Temporal trend analysis was performed to reveal increasing or decreasing trend over the 20-years period of research.
Description of data collection	Sediment samples were obtained by means of a stainless steel box and gravity corer operating on board of the R/V Aegaeo. The samples were analyzed for grain-size, organic carbon and major and trace elements contents with the same analytical techniques (sieving and X-ray absorption, CHN analyzer, XRF, respectively). Statistical analysis, including the Kolmogorov-Smirnov normality test and Factor analysis on Box-Cox and z-score transformed data was carried out using Principal Factor Analysis with Varimax rotation (XLSTAT). Enrichment factors were calculated using as a background the local, pre-industrial levels, previously established by using radio-chronology data. A multi-elemental pollution index was selected after reviewing the recent literature and employed to the most recently obtained data set to assess the current environmental status. Data of stations with at least a 5-year record (n = 14) were introduced to the MAKESENS software to assess whether temporal, increasing or decreasing trends of MPI exist.
Data source location	Saronikos Gulf, Greece, E. Mediterranean Sea Latitude and longitude for collected samples are given in Tables S1 & S2
Data accessibility	Data available within the article
Related research article	Aristomenis P. Karageorgis, Fotini Botsou, Helen Kaberi, Stylianos Iliakis, Geochemistry of major and trace elements in surface sediments of the Saronikos Gulf (Greece): assessment of contamination between 1999 and 2018, Science of the Total Environment, https://doi.org/10.1016/j.scitotenv.2020.137046 [1]

**Table:** 

**Value of the Data** • The dataset comprises the first detailed open access record of major and trace elements in the sediments of one of the most important, in terms of pollution, coastal area in the Eastern Mediterranean. • Data can assist researchers, policy makers, and stakeholders to better understand the coastal and open-sea environment of the area, support future management plans, and help to evaluate the effectiveness of current and future legislative tools and remediation effort. • Data could be combined with other indicators (hazardous organic and inorganic substances in sediments, biota and water) to assess the overall quality status of the coastal and marine environment within the frameworks of WFD and MSFD. • The dataset contributes to efforts of closing knowledge gap on metal levels in the Eastern Mediterranean Sea and could assist the development of the assessment criteria (background and background assessment concentrations) of Europe's regional seas.

## 1 Data description

**Fig. 1:**
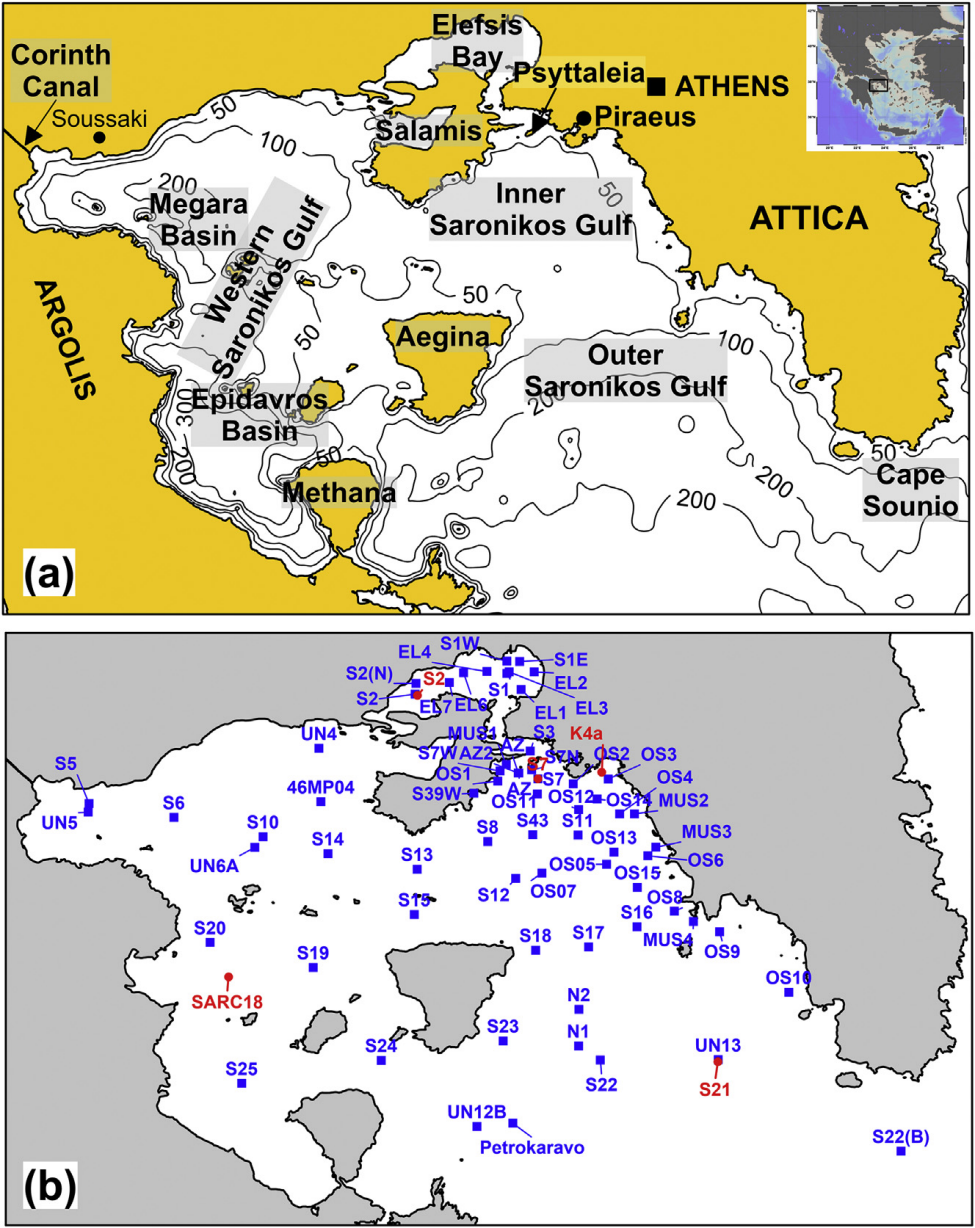
(a) Location map and bathymetry of the study area (depth contours in meters); (b) map showing the location of sediment sampling stations occupied from 1999 to 2018 (total of 216 samples obtained at 68 stations in blue color). The location of five cores used for metal backgrounds are illustrated in red color (S2, S7, S21, K4a, SARC18).

Surface sediments (0–1 cm) were collected from 68 sampling sites in the Saronikos Gulf and its sub-basins, namely, the Elefsis Bay, the Western (or Megara and Epidavros basin), the Inner and Outer Saronikos Gulf (Fig. 1a). Sampling was conducted by using a stainless steel box corer during thirteen oceanographic cruises of the R/V Aegaeo, from February 1999 to January 2018. A total of 216 samples were collected by using plastic tools and containers to avoid metal contamination. Several samples were obtained over the same network of stations serving the diachronic monitoring of the Saronikos Gulf with respect to major and trace elements contents. These samples were used to assess temporal trends of contaminants. Table S1 presents the grain-size, organic carbon, and major and trace elements contents of the surface sediments collected and analyzed from 1999 to 2018. The most recent dataset, *i.e.* the latest sediment samples (n = 68) obtained throughout the sampling network of Fig. 1b, is given in Table S2. The latter dataset was studied in more detail to gain insight of the current environmental status of the study area. It consisted of samples collected from the Western Saronikos Gulf in 1999 (n = 14); samples from the Elefsis Bay, the Inner and Outer Saronikos Gulf collected mostly from 2016 to 2018 (52 samples); two more samples were collected in the latter area in 2012–2013.

**Fig. 2:**
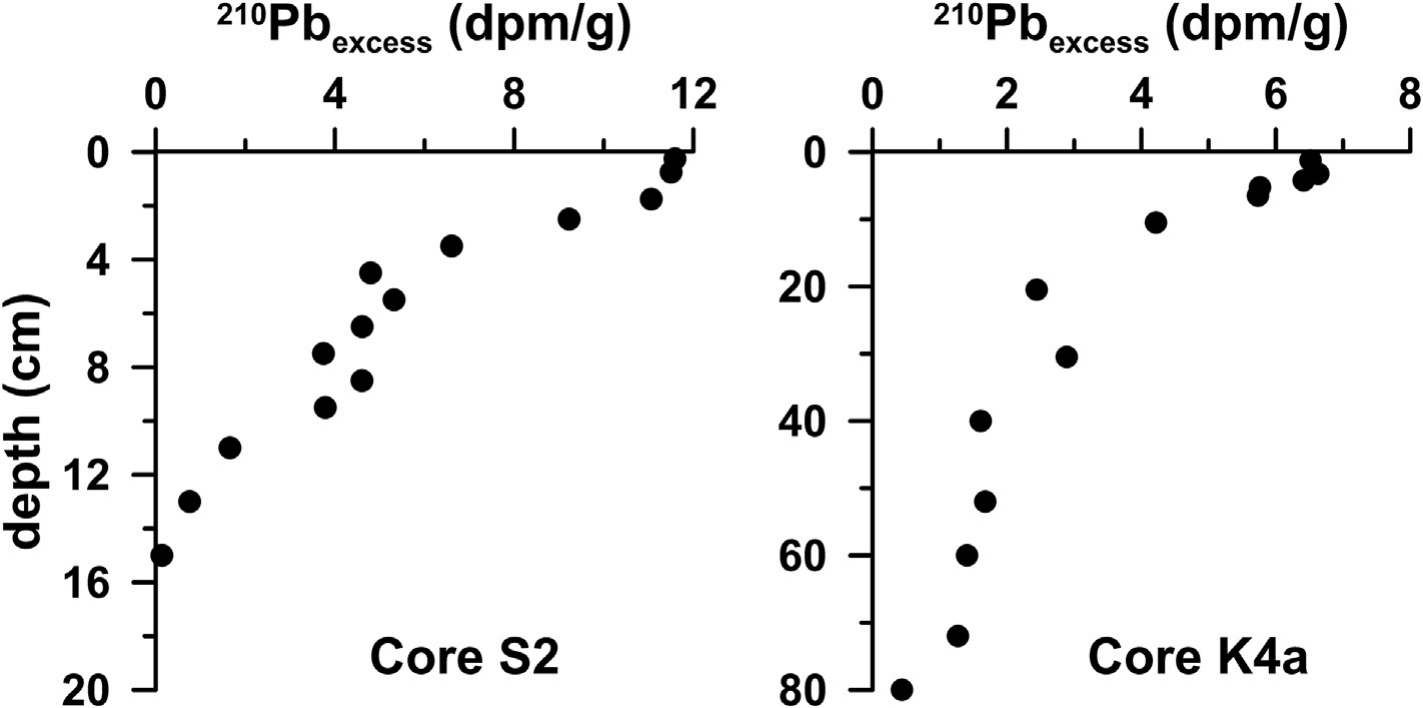
Down-core variation of ^210^Pb_excess_ for cores S2 and K4a.

**Fig. 3:**
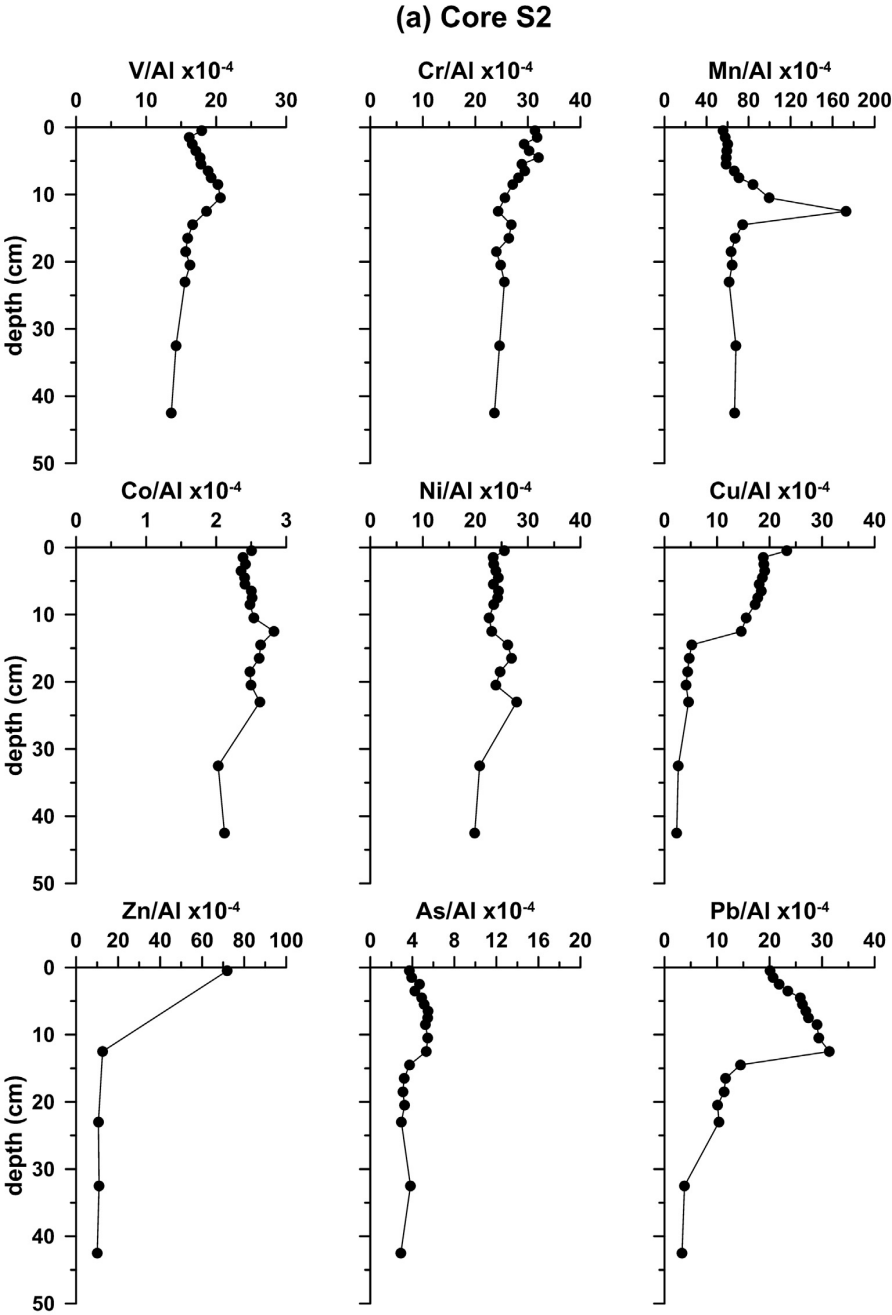

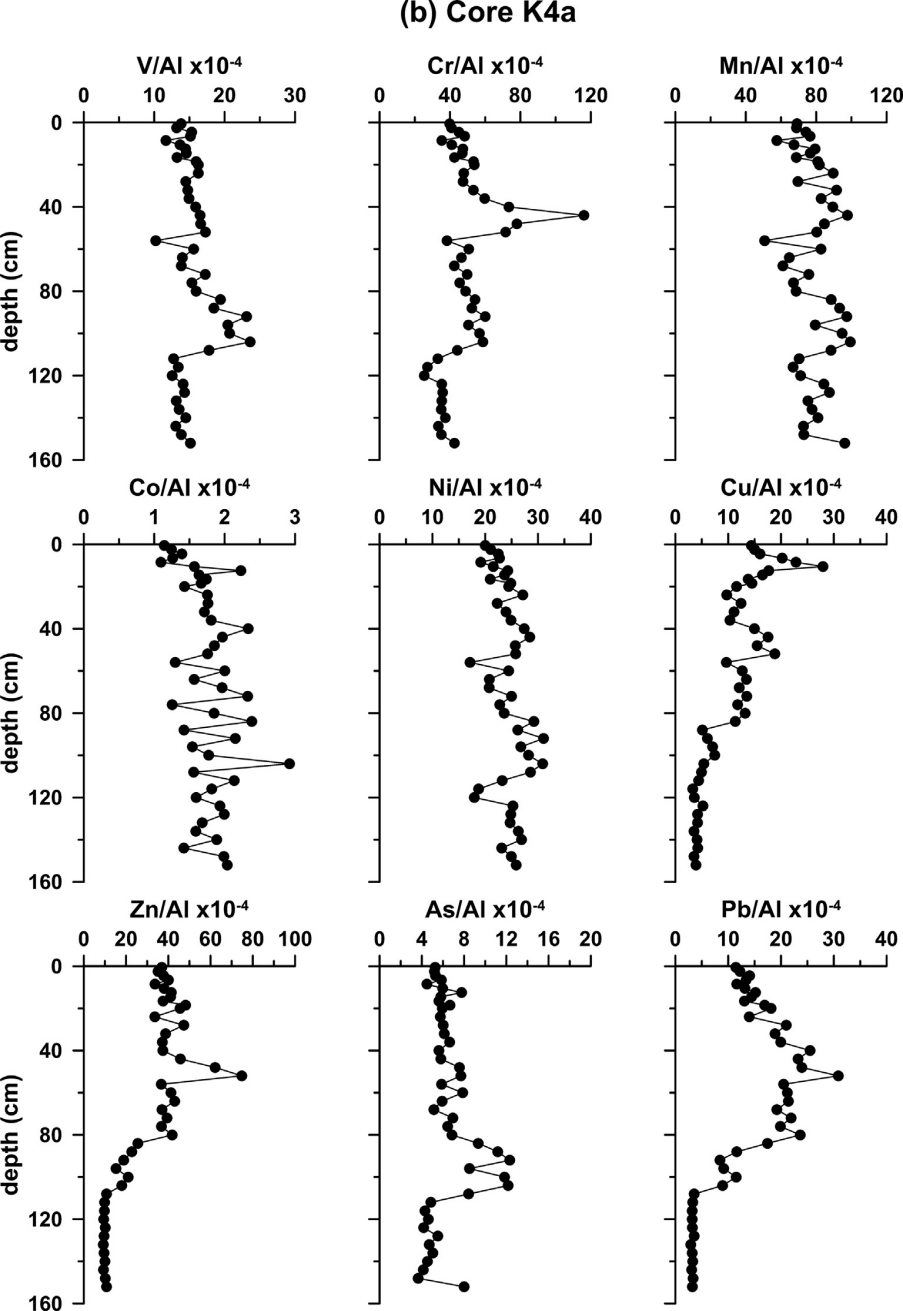

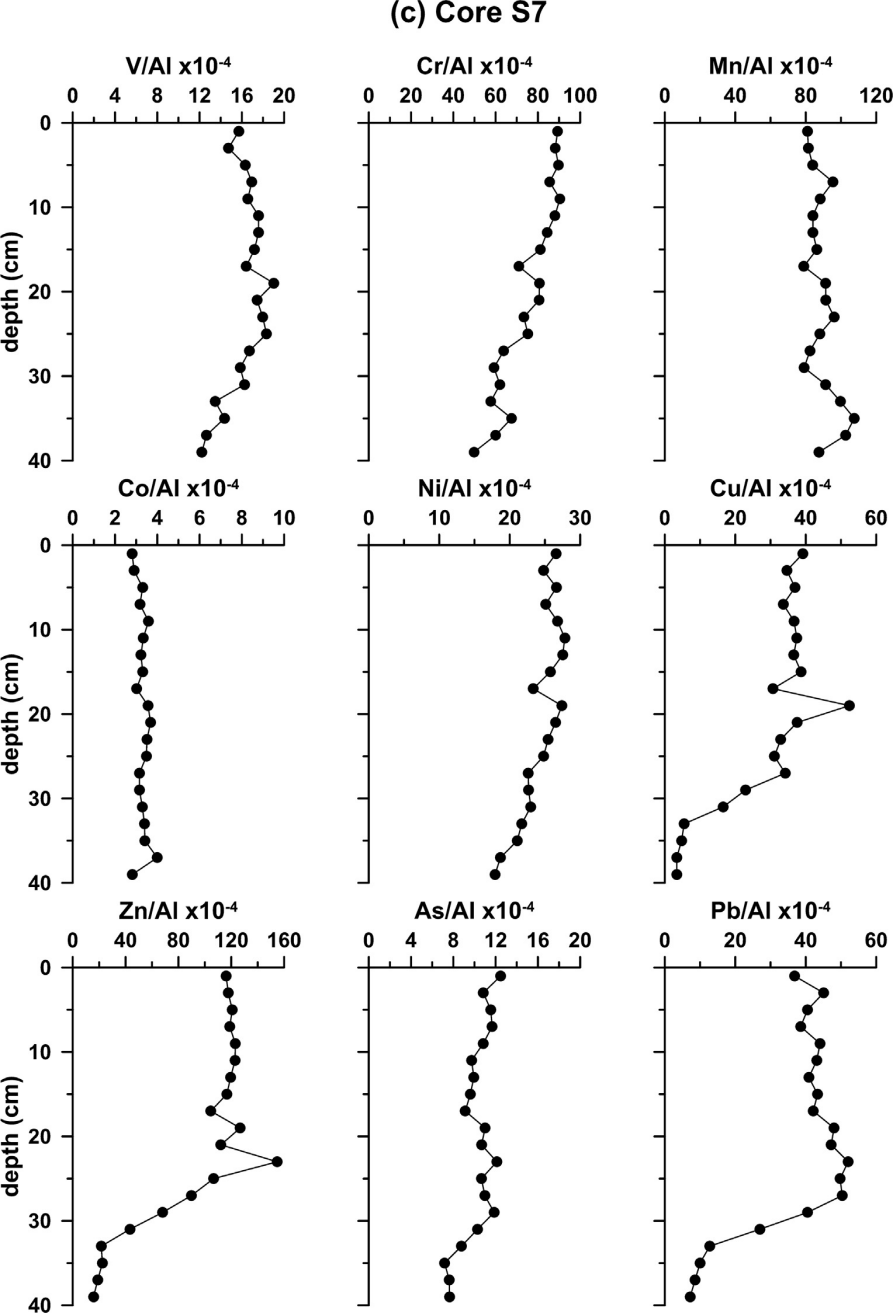

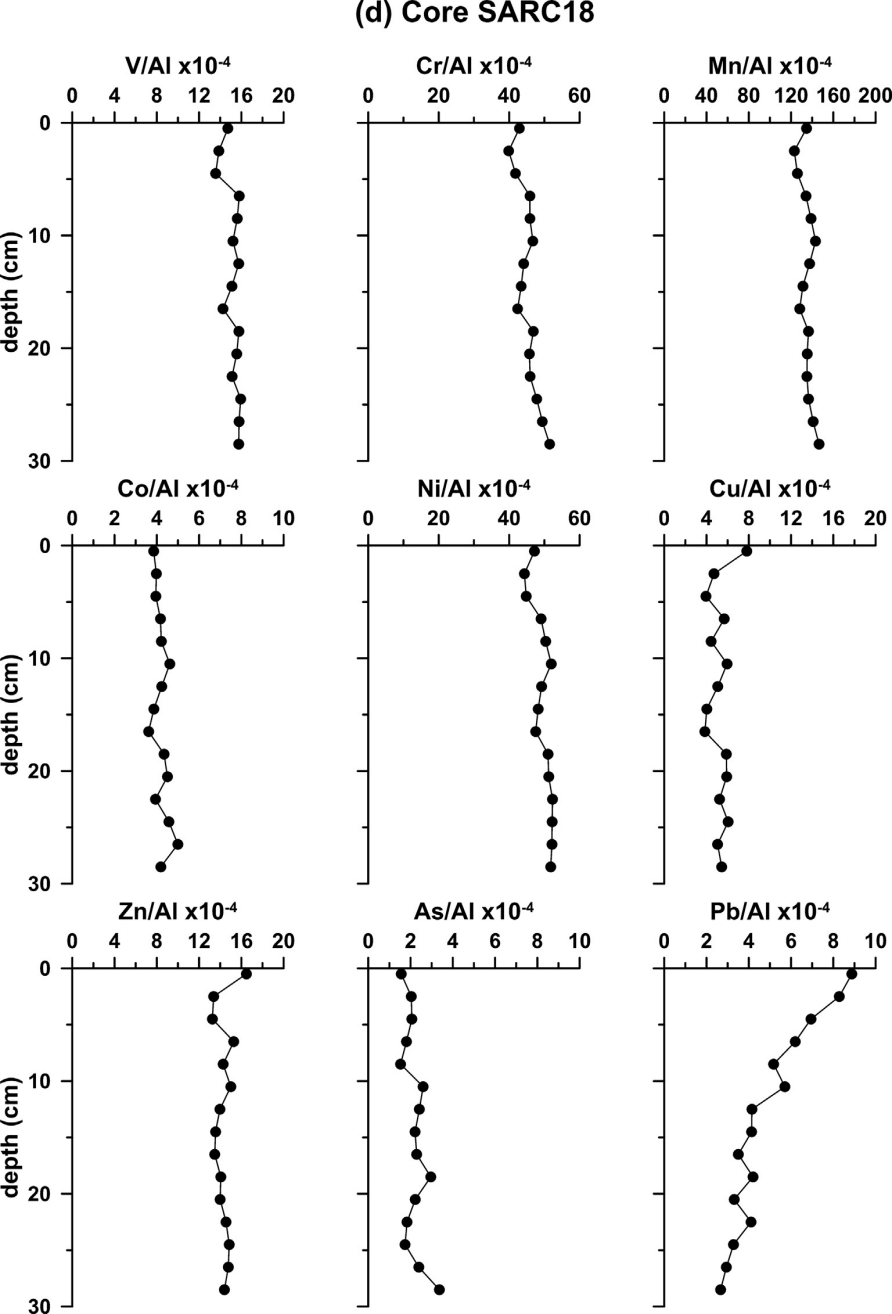

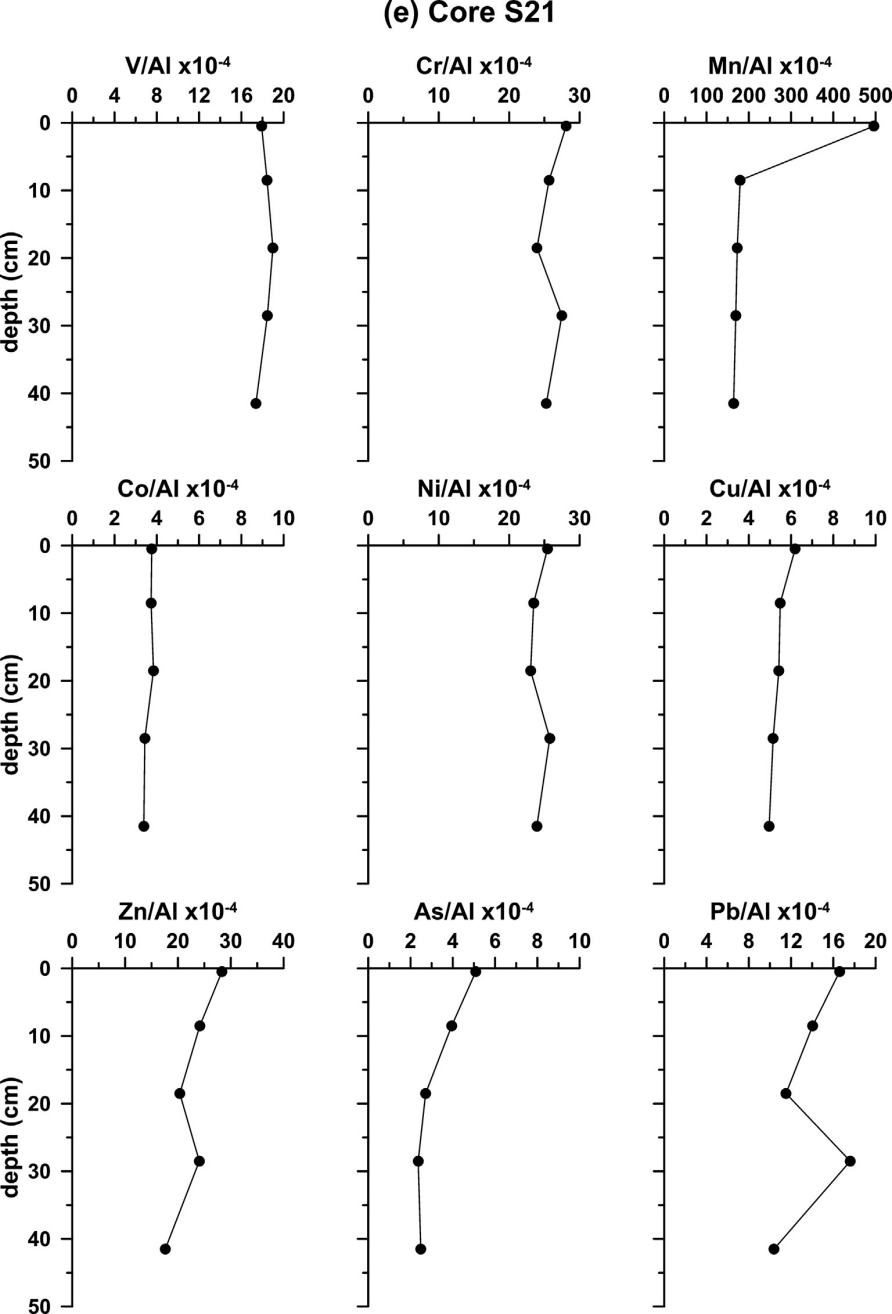
Down-core variability of element to Al ratios in cores (a) S2; (b) K4a; (c) S7; (d) SARC18; and (e) S21.

Furthermore, sediment cores were obtained from five locations. The K4a, S7 and SARC18 cores (Fig. 1b) were retrieved using gravity corer, whereas, the S2 and S21 using a box corer. Sediment cores aimed at establishing the local background levels of major and trace elements. The recent sedimentation rates in two cores, S2 and K4a, were calculated using the ^210^Pb method. The profiles of ^210^Pb_excess_ are shown in Fig. 2. The rest of the cores were dated by ^14^C accelerator mass spectrometer (AMS) analyses. The profiles of metal contents normalized to Al for the five sediment cores, collected from the four sub-sectors of the Saronikos Gulf are shown in Fig. 3.

**Fig. 4:**
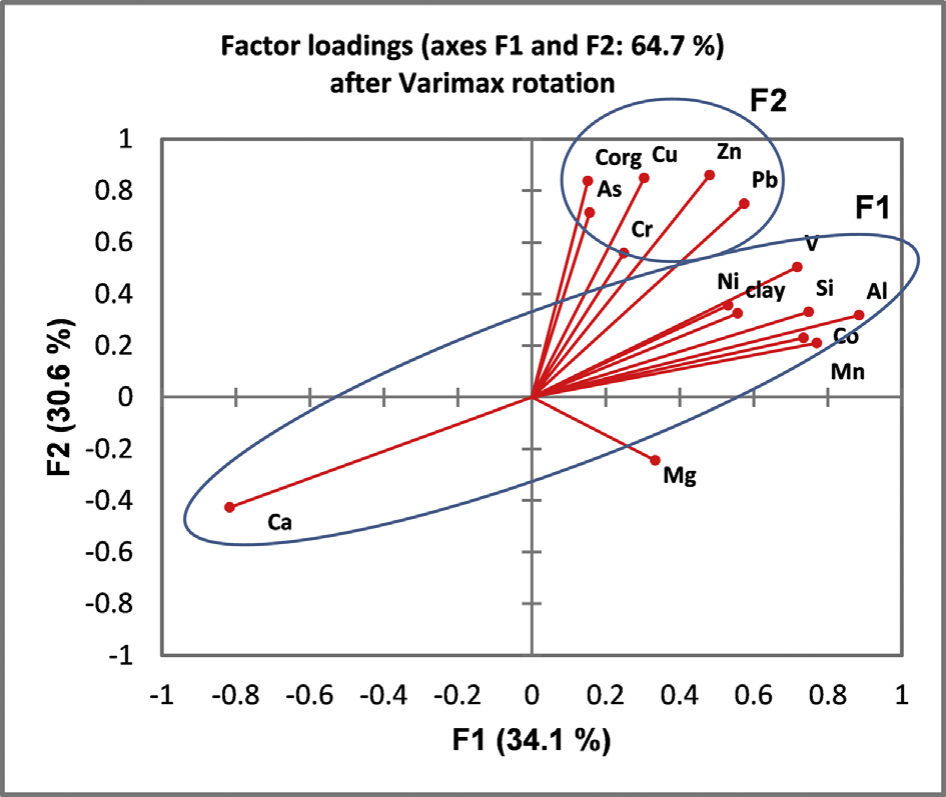
Factor plot after Varimax rotation.

Chemometric analysis was conducted in the most recently obtained sediment samples (n = 68) in order to explore inter-variable relationships and underlying common sources, as well as transport and deposition patterns. The variables of the dataset were first screened for normal distribution by the Kolmogorov-Smirnov (KS) normality test [2]. These results are given in Table 1. Transformed data to fulfil the requirements of normality [2,3], were introduced to Factor Analysis (FA). The analysis revealed three factors that explained 84% of the total variance (Table 2; Fig. 4). The first factor (34.1%) showed positive loadings for clay, Si, Al, V, Mn, Co; F2 (30.6%) exhibited high positive loading for C_org_, Cu, Zn, As, and Pb; F3 (19.3%) associated Mg, Cr, Ni, Co, V, and Mn.

**Table 1:** Results of Kolmogorov–Smirnov test for normality of the original (*p*), the log-transformed (*p*_log), and the Box–Cox/*z*-transformed (*p*_tr) data.

	p	*p*_log	*p*_tr
sand	0.022^a^	0.016^a^	0.044^a^
silt	0.101	0.010^a^	0.027^a^
clay	0.025^a^	0.282	0.163
C_org_	0.006^a^	0.599	0.527
Si	0.296	0.033^a^	0.365
Al	0.270	0.153	0.532
Ca	0.698	0.601	0.726
Mg	0.226	0.830	0.854
V	0.068	0.514	0.525
Cr	0.572	0.109	0.412
Mn	<0.0001	0.835	0.868
Co	0.287	0.310	0.661
Ni	0.04^a^	0.915	0.858
Cu	0.010^a^	0.984	0.952
Zn	0.001^a^	0.833	0.911
As	0.091	0.459	0.760
Pb	0.023^a^	0.959	0.961

a Reject the normality hypothesis at a 0.05 significance level.

**Table 2:** Factor pattern after Varimax rotation.

Variable	F1	F2	F3
clay	**0.556**	0.325	0.552
C_org_	0.151	**0.837**	0.081
Si	**0.748**	0.331	0.317
Al	**0.884**	0.319	0.280
Ca	**−0.817**	−0.427	−0.283
Mg	0.334	−0.244	**0.544**
V	**0.717**	0.505	0.452
Cr	0.249	0.559	**0.748**
Mn	**0.770**	0.210	0.469
Co	**0.735**	0.229	0.595
Ni	0.531	0.357	**0.768**
Cu	0.304	**0.849**	0.215
Zn	0.481	**0.860**	0.170
As	0.156	**0.714**	0.072
Pb	0.574	**0.750**	0.159

Values in bold correspond for each variable to the factor for which the squared cosine is the largest.

A two-step approach was followed for assessing pollution. First, Enrichment Factors (EFs) for the recent dataset of surface sediments were calculated according to Eq. (1):

(1) $$\text{EF}={\left(\text{element/Al}\right)}_{\text{sample}}\text{/}{\left(\text{element/Al}\right)}_{\text{background}}$$

where background refers to the local pre-industrial levels established for each sub-sector of the study area by taking into account the results of ^14^C dating and the recent sedimentation rates reported in Ref. [1]. The calculated EFs are presented in Table 3.

**Table 3:** Enrichment factors for trace elements and Modified Pollution Index estimated for the most recent sediment samples (n = 68) of the Saronikos Gulf and the Elefsis Bay.

Year-Month	Station	EF V	EF Cr	EF Mn	EF Co	EF Ni	EF Cu	EF Zn	EF As	EF Pb	MPI
**1999**	S5	1.25	1.96	1.31	1.47	1.93	0.82	1.19	1.40	1.87	1.7
	S6	1.11	1.15	2.76	1.07	1.16	1.02	1.54	1.54	2.81	2.3
	S10	1.12	1.12	1.23	0.86	0.95	1.09	1.46	1.50	2.73	2.1
	S12	1.33	0.80	1.58	1.25	1.36	1.74	1.99	0.35	5.66	4.2
	S14	0.52	0.73	0.69	0.95	0.36	0.48	0.96	2.01	2.41	1.9
	S15	0.32	0.45	0.55	0.59	0.21	0.33	0.76	2.96	2.01	2.2
	S17	0.49	0.89	1.22	0.81	0.57	1.23	1.15	1.78	1.27	1.5
	S18	0.30	0.62	0.62	0.53	0.38	0.69	0.79	1.40	0.95	1.1
	S19	0.38	0.42	0.47	0.64	0.19	0.36	0.55	1.99	1.66	1.5
	S20	1.12	1.10	1.67	0.97	1.06	1.19	1.50	1.45	2.48	2.0
	S22	1.02	1.35	3.10	1.15	1.10	1.27	1.62	1.89	1.36	2.4
	S23	0.45	0.48	0.93	0.59	0.26	0.50	0.55	1.08	0.63	0.9
	S24										
	46MP04	1.21	1.85	1.36	0.94	0.96	1.19	1.58	2.13	3.01	2.4
**2012**	S39W	0.78	0.83	1.03	0.52	0.73	2.01	1.70	0.37	3.14	2.4
**2013**	Petrokaravo	0.99	1.46	1.54	1.03	1.03	1.25	1.26	0.94	1.02	1.4
**2016**	S2(N)	1.40	1.96	2.32	1.61	1.99	10.60	6.06	1.69	4.71	7.9
	S7W	0.92	1.30	1.03	0.82	1.21	3.11	2.42	0.77	4.72	3.6
	S7N	1.13	1.43	1.15	1.07	1.46	5.82	4.43	0.81	10.63	7.8
**2017–03**	S25	1.14	0.83	9.17	1.15	0.83	1.66	1.64	1.21	2.90	6.7
**2017–09**	OS9	0.82	2.05	0.88	0.95	0.87	0.62	1.73	2.79	1.78	2.2
	OS10	0.78	2.16	0.99	0.88	0.77	1.73	2.15	6.70	3.76	5.0
	AZ	1.57	2.19	1.61	1.60	2.11	4.76	6.01	1.13	10.75	8.0
**2017–10**	N1	0.63	2.05	1.17	1.47	1.57	1.42	1.02	1.93	0.84	1.7
	N2	0.80	1.41	1.19	1.22	1.05	1.04	1.54	1.81	1.53	1.6
	S22(B)	0.79	2.28	1.26	1.23	0.87	0.91	1.72	3.34	1.61	2.6
	UN4	0.99	2.94	1.36	1.33	1.25	1.20	1.69	3.20	2.70	2.6
	UN5	1.06	2.09	2.27	1.49	2.03	1.21	1.51	1.63	2.78	2.3
	UN6A	0.83	1.11	2.13	1.10	1.06	1.33	1.38	1.31	2.82	2.2
	UN12B	0.80	1.30	1.65	0.99	1.07	1.01	1.23	1.25	1.09	1.4
	UN13 (S21)	0.80	1.24	1.67	1.13	1.04	1.09	1.47	1.61	1.34	1.5
	EL1	1.31	1.82	1.22	1.23	0.87	11.96	12.40	1.76	11.69	9.4
	EL2	0.96	1.29	2.48	1.00	0.58	3.72	5.36	1.55	4.83	4.2
	EL3	1.27	1.43	1.61	1.20	1.05	8.13	7.05	0.80	7.41	6.2
	EL4	1.10	1.22	1.35	1.21	0.76	4.82	4.43	1.06	4.48	3.8
	EL6	1.05	0.95	0.95	1.04	0.83	3.11	7.12	1.21	7.17	5.4
	EL7										
	OS05	1.95	1.72	4.34	2.41	2.26	4.86	4.75	1.52	12.88	9.6
**2017–11**	OS07	1.23	0.92	2.24	1.51	1.68	1.60	2.03	0.37	6.11	4.5
	AZ	1.79	2.38	1.70	0.92	2.13	8.51	8.89	1.24	16.64	12.3
	S1	1.19	1.26	3.07	1.27	0.91	7.79	6.76	1.27	7.49	6.0
**2018–01**	S1W	1.08	1.09	2.80	1.22	1.25	1.71	3.96	0.99	5.31	4.0
	S1E	1.07	1.06	2.95	1.36	1.18	2.31	4.18	1.05	5.35	4.1
	S2	1.44	1.64	4.02	1.48	1.77	7.39	6.33	1.58	5.16	5.8
	S3	1.13	1.34	1.21	0.83	1.30	9.05	7.27	0.82	15.38	11.3
	S7	1.43	1.61	1.09	1.12	1.63	9.72	9.04	1.09	20.65	15.1
	S8	0.89	0.68	1.16	0.88	1.13	1.67	1.56	0.26	4.35	3.2
	S11	0.84	0.76	2.10	1.34	1.23	3.29	2.20	1.32	7.46	5.5
	S13	0.63	0.99	1.65	1.09	0.53	1.45	1.87	3.92	3.35	3.0
	S16	0.63	0.64	2.41	0.99	0.90	0.87	1.83	0.43	6.33	4.6
	S43	1.02	0.75	1.55	1.00	1.39	1.99	1.66	0.28	4.64	3.5
	OS1	1.30	2.00	1.81	1.57	1.65	6.53	5.40	0.97	13.27	9.8
	OS2	1.24	0.98	0.95	1.02	1.34	4.49	3.38	0.65	8.14	6.0
	OS3	1.26	1.47	1.82	1.29	1.09	2.79	2.83	0.86	6.52	4.9
	OS4	0.87	0.93	1.33	0.73	0.88	1.02	1.83	0.80	4.54	3.4
	OS6	1.86	1.74	2.23	1.66	1.70	0.00	3.09	2.16	8.95	6.6
	OS8	0.85	0.91	1.71	1.17	0.96	1.36	2.06	1.13	5.41	4.0
	OS11	1.08	0.91	1.18	0.87	1.32	3.47	2.53	0.45	7.23	5.3
	OS12	1.06	1.21	1.96	1.74	1.06	2.97	2.41	0.70	7.21	5.3
	OS13	2.08	1.74	2.35	1.58	1.89	3.45	3.96	1.96	11.10	8.2
	OS14	1.47	1.28	1.38	1.23	1.33	3.34	3.48	0.88	7.61	5.7
	OS15	0.86	0.68	1.56	0.00	0.69	3.51	1.75	1.27	3.31	2.7
	AZ2	1.56	2.44	1.81	2.05	2.14	8.94	8.18	1.62	16.12	11.9
	SEL1	1.39	0.63	2.35	1.09	0.97	3.72	4.97	0.41	8.60	6.4
	MUS1	3.25	9.57	5.38	4.90	4.74	10.08	9.03	4.58	18.97	14.5
	MUS2	1.68	2.65	3.54	2.70	2.38	7.83	4.16	2.66	9.98	7.7
	MUS3	0.69	0.72	0.92	0.74	0.68	2.34	1.29	0.73	3.17	2.4
	MUS4	2.85	2.73	4.98	3.92	4.44	7.66	4.82	2.35	14.21	10.7

As a second step for assessing contamination, the multi-elemental Modified Pollution Index (MPI), introduced by Brady et al. [4] was used. The calculated MPI values are given in Table 3.

(2) $$MPI=\sqrt{\frac{{\left(EF_{mean}\right)}^{2}+{\left(EF_{max}\right)}^{2}}{2}}$$

Trend analysis was conducted to identify significant and sustained, increasing or decreasing trends of the MPI. Trend analysis was performed using the sampling sites with available data for more than 5 years, totaling 14 sites. Table 4 presents the output of the model.

**Table 4:** Results of the Mann-Kendall test and Sen's magnitude of slope using the MAKESENS software [11].

Station	Time series			Mann-Kendall trend		Sen's slope estimate	
	First year	Last Year	n	Test S	Signific.	Q	B
S1	1999	2018	9	−22	∗	−0.260	18.515
S1E	2010	2018	5	2		0.369	4.453
S1W	2009	2016	5	−2		−0.549	8.824
S2	1999	2018	9	−22	∗	−0.063	3.691
S3	2001	2018	8	−12		−1.259	31.726
S7	1999	2018	9	0		0.001	2.574
S7 W	2009	2016	5	−2		−0.549	8.824
S7N	2009	2016	5	−4		−0.984	16.157
S8	1999	2018	8	−6		−0.056	4.374
S11	1999	2018	8	−6		−0.258	8.024
S13	1999	2018	8	−2		−0.013	3.408
S16	1999	2018	8	2		0.040	5.820
S26	2009	2017	6	−3		−0.036	6.032
S43	2001	2018	5	−2		−0.014	3.690

## 2 Experimental design, materials, and methods

### 2.1 Grain-size

Samples were wet sieved through a 63 μm stainless steel mesh. The finer fraction was analyzed with a Micromeritics Sedigraph 5100E, whereas samples collected after March 2017 with a Micromeritics III *PLUS*, in order to separate silt and clay fractions. Calgon (5.5 g L^−1^) and sonication (60 s) was used for disaggregation/dispersion. Subsequently, the dry percentages of sand (>63 μm), silt (2 μm <Ø <63 μm), and clay (<2 μm) were calculated and the nomenclature followed Folk [5].

### 2.2 Organic carbon content

Sediment samples were thoroughly ground in an agate mortar and very well homogenized to reduce variability between replicates. Splits of 10–20 mg of powdered homogenized sample were weighed accurately (0.01 mg) into specially designed silver containers. Organic carbon was determined after removal of inorganic carbon by acidification of samples with 20 μL of 6 N HCl at 60 °C (this treatment was conducted five times in 12 hours intervals). After the inorganic carbon removal, the samples were dried at 60 °C overnight. Then, the containers were pinched closed, compacted, and formed into a ball. The balls then were placed in the auto-sampler of a Fisons Instruments CHN elemental analyzer type EA-1108 to determine organic carbon contents. The operating parameters were very similar to those reported in Refs. [[6], [7], [8]]. The precision of the method was within 5%.

### 2.3 Major and trace elements

Samples were sieved through a 1 mm sieve, oven dried at 40 °C to remove moisture, and then ground to a fine powder in a motorized mill with agate mortar and balls. Sediment samples were analyzed for their chemical composition in a PANalytical (former Philips) PW-2400 wavelength X-Ray Fluorescence analyzer, equipped with Rh-tube. Major elements were determined in fused beads (SiO_2_, Al_2_O_3_, TiO_2_, Fe_2_O_3_, K_2_O, Na_2_O, CaO, MgO, P_2_O_5_); for the purposes of the present paper we present only Si, Al, Mg and Ca contents and corresponding spatial distributions. Fused bead preparation involved a complete fusion of 0.6 g of sample, with 5.4 g of flux (50:50 lithium *meta*-borate, lithium tetra-borate) and 0.5 g of lithium nitrate, the latter being used as an oxidizer. Loss on ignition (LOI) was determined after burning 1 g of sample for 1 h at 1000 °C.

Trace elements were determined according to the following procedure: 5 g of powdered sample were mixed with 1.25 g of wax and subsequently pressed in a 31 mm aluminum cup (20 s, 20 tons). The powder pellets were analyzed in the XRF to determine trace element contents (V, Cr, Mn, Co, Ni, Cu, Zn, As, Pb) using PANalyticals's Pro-Trace reference sample set and software for the instrument calibration. Analyses was based on 2-point calibrations forced through the origin, using 25 multi-element high quality standards and two blank samples, which allow the XRF system calibration for elements ranging from Sc to U; background, line overlap, and matrix corrections were applied. Analytical accuracy was checked by parallel analysis of the certified sediment standard PACS-2 and was found to be better than 7% for all elements analyzed. Analytical precision was checked in sample replicates and was always better than 0.5%. Detection limits were below 5 mg kg^−1^ (V, Co, Ni, As), 5–10 mg kg^−1^ (Cr, Cu, Pb), and 10–12 mg kg^−1^ (Mn, Zn) for the elements determined (see also Table S1). To check long-term repeatability in the framework of the present study, archived powder samples were re-scanned.

### 2.4 Recent sedimentation rates

For the calculation of the recent sedimentation rates, the down core total ^210^Pb activity was determined through the activity of its alpha-emitting granddaughter ^210^Po, assuming secular equilibrium with ^210^Pb. For the total dissolution of the dried sediments the analytical method described by Sanchez-Cabeza et al. [9] was applied. The sedimentation rates were calculated using the widely used Constant Rate of Supply model (CRS) [10].

### 2.5 Factor analysis

Factor analysis is a powerful method in geochemistry that explains the variation in a multivariate data set by a limited number of factors [2], provided that certain precautions are taken. The data set (most recent data set; n = 68) was carefully checked for outliers by means of Box-and-Whisker plots, and a few stations were removed from FA analysis due to the presence of several univariate outliers. Since FA is based on the correlation (or covariance) matrix, it is sensitive to non-normally distributed variables, thus various transformations are required prior to analysis [2]. Normal distribution of all variables was tested by a Kolmogorov-Smirnov (KS) normality test. Eight out of 18 variables did not pass the KS test run on the original data, whereas four variables did not pass the KS test run on log-normalized data (Table 2). A Box-Cox transformation followed by a z-transformation [3] provided the best results, where all variables but sand, and silt passed the KS test; grain-size variations were therefore explained by the clay fraction variability. Subsequently, Principal Factor Analysis with Varimax rotation was conducted in order to group clay, organic carbon and major and trace elements.

### 2.6 Assessment of temporal trends

The MAKESENS program has been developed by the Finnish Meteorological Institute [11] for detecting trends of annual values of atmospheric pollutants. MAKESENS performs two types of statistical analyses: First, the presence of a monotonic, increasing or decreasing trend is tested with the nonparametric Mann-Kendall test, and second, the slope of a linear trend is estimated with the nonparametric Sen's method [12].

The Mann-Kendall test is applicable in cases when the data values x_i_ of a time series can be assumed to obey the model:

(3) $$x_{i}=f\left(t_{i}\right)+\varepsilon_{i}$$

where:

f(t) is a continuous monotonic increasing or decreasing function of time, and ε_i_ are the residuals that can be assumed to be from the same distribution with zero mean.

The null hypothesis H_0_ is that observations *x*_*i*_ display no trend and is tested against the alternative hypothesis H_1_ that there is an increasing or decreasing monotonic trend. For time series with less than 10 observations (as in this study), the *S* statistics is calculated by the formula [12]:

(4) $$S=\sum_{k=1}^{n-1}\sum_{j=k+1}^{n}sgn\left(x_{j}-x_{k}\right)$$

where:

*n* is the number of annual values in the studied data series, *x*_*j*_ and *x*_*k*_ are the annual values in years *j* and *k*, respectively, and *j > k*, and

(5) $$sgn\left(x_{j}-x_{j}\right)=\{\begin{array}{c} 1\;if\;x_{j}-x_{k}>0\\ 0\;if\;x_{j}-x_{k}=0\\ -1\;x_{j}-x_{k}<0 \end{array}$$

For n ≤ 9 the absolute value of *S* is compared to the theoretical distribution of *S* derived by Mann and Kendall [12]. In MAKESENS the two-tailed test is used for four different significance levels *α*: 0.1, 0.05, 0.01 and 0.001. At certain probability level H_0_ is rejected in favour of H_1_ if the absolute value of *S* equals or exceeds a specified value *S*_*α/2*_, where *S*_*α/2*_ is the smallest *S* which has the probability less than *α/2* to appear in case of no trend. A positive value of *S* indicates an upward trend, and a negative value indicates a downward trend [11].

The non-parametric Sen's method is used to estimate the true slope of an existing trend. It can used in cases where the trend can be assumed to be linear. Thus, the *f(t)* in Eq. (3) could be written as:

(6) f(t)=Qt+B

where *Q* is the slope and *B* is the constant.

To calculate the slope estimate *Q* of Eq. (6), the slopes of pairs of data values are calculated first:

(7) $$Q=\frac{x_{j}-x_{k}}{j-k}$$

where *j > k.*

For *n* values of *x*_*j*_ and *N* data pairs for which *j > k*, the Sen's estimator of slope will be the median of these *N* values of *Q*.

The constant *B* of Eq. (6) is the median of the n values of differences *x*_*i*_ *– Q*_*ti*_.
